# Systemic lupus erythematosus is associated with an increased risk for orthorexia nervosa: a cross-sectional study

**DOI:** 10.1007/s00296-026-06107-2

**Published:** 2026-04-01

**Authors:** Eleni C. Pardali, Katerina Maria Kontouli, Arriana Gkouvi, Myrto Samara, Odysseas Androutsos, Christina G. Katsiari, Dimitrios G. Goulis, Dimitrios P. Bogdanos, Maria G. Grammatikopoulou

**Affiliations:** 1 Immunonutrition Unit, Department of Rheumatology and Clinical Immunology, Faculty of Medicine, School of Health Sciences, University of Thessaly, GR-41223, Biopolis, Larissa, Greece; 2 Laboratory of Hygiene and Epidemiology, Faculty of Medicine, University of Thessaly, Larissa, Greece; 3 Department of Primary Education, School of Education, University of Ioannina, Ioannina, Greece; 4 Department of Psychiatry, Faculty of Medicine, University of Thessaly, Larissa, Greece; 5 Laboratory of Clinical Nutrition and Dietetics, Department of Nutrition and Dietetics, School of Physical Education, Sports Science and Dietetics, University of Thessaly, 42100 Trikala, Greece; 6 Unit of Reproductive Endocrinology, 1st Department of Obstetrics and Gynecology, Aristotle University of Thessaloniki, Thessaloniki, 56429 Greece

**Keywords:** Diet, Nutrition therapy, Feeding and eating disorders, Systemic lupus erythematosus, Fibromyalgia, Surveys and questionnaires, Feeding behavior, Avoidant/restrictive food intake disorder

## Abstract

Orthorexia nervosa (ON) is an atypical eating disorder marked by persistent preoccupation with consuming only ‘pure’ or healthy foods, coupled with harmful compulsive behaviors. Rheumatic and musculoskeletal diseases (RMDs) are a broad group of conditions, characterized by pain and inflammation, affecting the joints and other connective tissues. Nutritional factors have been shown to exert a substantial influence on the development of these pathologies; however, orthorexic tendencies have not yet been studied among RMDs. This cross-sectional study recruited 367 patients from the Department of Rheumatology and Clinical Immunology at the University General Hospital, situated in Larissa. Patients were interviewed during their regular appointments in order to assess orthorexic tendencies. ON was evaluated using the Düsseldorf Orthorexia Scale (DOS), and linear regression analyses were performed. Although the presence of ON was relatively uncommon among patients with RMDs (2.2%), women (β = 2.41, 95% CI: 1.16, 3.65, *p* = 0.0002) and patients with systemic lupus erythematosus (β = 2.01, 95% CI: 0.39, 3.64, *p* = 0.02) or fibromyalgia syndrome (β = 8.17, 95% CI: 2.61, 13.73, *p* = 0.004) were at a greater risk for ON. On the other hand, patients with idiopathic inflammatory myopathies demonstrated a reduced propensity for orthorexic tendencies (β=-2.39, 95% CI: -4.70, -0.09, *p* = 0.04). Although ON demonstrates a low prevalence among patients with RMDs, certain diagnoses are associated with a higher propensity for orthorexia. Identifying ON is important for improved disease outcomes, metabolic function, and patients’ quality of life.

## Introduction

Orthorexia Nervosa (ON) is currently regarded as an unspecified feeding and eating disorder (USFED) [1] and is not formally recognized as a distinct diagnosis in the *Diagnostic and Statistical Manual of Mental Disorders* (DSM‑5) or the *International Classification of Diseases*,* 11th edition* [2]. Although not yet an official entity, ON has been linked to significant underlying psychopathology and exhibits notable symptom overlap with several conditions including anorexia nervosa (AN), obsessive–compulsive disorder (OCD), somatic symptom disorder, illness anxiety disorder, and psychotic spectrum disorders [3]. ON is characterized by persistent and intrusive thoughts regarding “clean” or “healthy” eating —its name deriving from the Greek ortho (correct) and orexis (appetite),— and by compulsive dietary behaviors driven by an ideology of “healthism” [4, 5]. These behaviors often involve extensive preoccupation with food selection, sourcing, preparation, and adherence to rigid self-imposed dietary rules, accompanied by considerable stress in striving to meet these standards [6]. Inevitably, such patterns can result in cognitive and emotional impairment caused by social and physical distancing, as well as micronutrient restriction, resulting in nutritional deficiencies and ultimately malnutrition [6]. Some authors have proposed that ON can be conceptualized within the framework of Avoidant/Restrictive Food Intake Disorder (ARFID), since both involve restrictive eating patterns [2]. However, ARFID is commonly linked to sensory sensitivities or fear of the consequences of eating, rather than an excessive fixation on health [7]. The lack of a standardized definition of ON also complicates its assessment, as no existing diagnostic tools can fully capture the nuances of orthorexic behavior. Several instruments have been proposed for the assessment of ON, including the Bratman Orthorexia Test and the ORTO-15, which are commonly used despite concerns for poor psychometric properties [3, 8] and their inability to differentiate between ON and overall health-conscious eating. More recent tools, such as the Düsseldorf Orthorexia Scale (DOS), have demonstrated stronger reliability and validity, though being self-reported [9].

Medical Nutrition Therapy (MNT), which includes nutritional support for patients with chronic diseases, is gaining attention as part of a more holistic approach to therapy [10]. However, adherence to prescribed dietary regimens can, in some cases, take an uncontrolled turn, extending well beyond healthy eating and evolving into healthism [11–13]. High rates of orthorexic tendencies have been reported in populations with chronic diseases, including up to 81.3% in patients with diabetes mellitus [11], 77% in those with inflammatory bowel disease [14], 71% in individuals with celiac disease [12] and 36.7% of women with cancer being identified as being at high risk for ON [15].

In autoimmune rheumatic and musculoskeletal disorders (RMDs), MNT is generally proposed to alleviate physical symptoms and act beneficially in several ways [16]. Autoimmune diseases are related to a greater risk of presenting a mood disorder [17], while a recent study suggests that more than half of patients with systemic autoimmune RMDs are suffering from depression and anxiety [18, 19]. Cross-sectional studies suggest that people with depression, anxiety and stress are more likely to present orthorexic tendencies [20–22]. Autoimmune RMDs constitute a group of disorders in which dysregulation of the immune system leads to loss of self-tolerance damaging the body’s own tissues [23]. Nutritional status exerts a substantial influence on the progression of the disease and associated morbidity [24, 25], a phenomenon that is recognized by a significant proportion of patients [26]. However, patients with RMDs are predominantly reported to opt for nutrient-inadequate diet patterns [27, 28] or adhere to a Mediterranean style diet [29]. A growing body of research has indicated the existence of a relationship between eating disorders and RMDs, including AN, bulimia nervosa, and ON [30–32]. Whether orthorexic tendencies are present in RMDs remains unclear, as does the role of contributing factors within this framework. The present study thus sought to shed light on such possible relationships.

## Materials and methods

### Study design and sample

This single-center, cross-sectional study was conducted at the Department of Rheumatology and Clinical Immunology situated at the Larissa University General Hospital, and included 367 consecutive patients with RMDs. Patients were recruited between February 2023 and May 2025 during their visits to the inpatient and outpatient clinics or while they attended scheduled intravenous immunotherapy sessions. The present study was approved by the Larissa Hospital Scientific Board (30/3rd/20-02-2025). The sample size was not calculated, as collected data corresponded to the maximum power of patients recruited from a specific hospital in a defined region. Patients who met the following criteria were included in the study: (i) patients diagnosed with at least one RMD, (ii) able to communicate effortlessly in the Greek language. Individuals diagnosed with concomitant cancer, those who were pregnant, and those aged below 18 years were excluded from the sample. All consecutive patients who met the inclusion criteria were recruited for the study. Table 1 presents details on the patients’ characteristics.

**Table 1:** Characteristics of the study population (*N* = 367)

Variables	Categories and Units	Values
Sex	Women/Men (*n*, %)	256 (69.8)/ 111 (30.2)
Age (yrs)	Women/Men (*n*, %)	61 (51–70)/ 61 (51–74)^†^
Education attained	Primary/Middle School/Tertiary/Postgraduate (*n*, %)	161 (43.9)/ 121 (33.0)/ 78 (21.3)/ 5 (1.4)
Employment status	Full-time/Part-time/Unemployed /Retired/Ill-health retirement (*n*, %)	90 (24.5)/17 (4.6)/ 67 (18.3)/ 161 (43.9)/ 31 (8.4)
Race	Caucasian/Roma/Latino/African American (*n*, %)	352 (95.9)/11 (3.0)/ 2 (0.5)/ 1 (0.3)
Current smokers	(*n*, %)	70 (19.1)
BMI (kg/m^2^)		26.95 (23.5–30.19)^†^
Weight status	Underweight/Normoweight/Overweight-Obese (*n*, %)	14 (3.8)/ 107 (29.3)/244 (66.8)
Recruitment	Inpatient/Outpatient/IV immunotherapy (*n*, %)	174 (47.4)/ 45 (12.3)/ 146 (39.8)
Current GC use	(*n*, %)	148 (40.3)
CRP (mg/dL)		0.6 (0.18–2.53)^†^
ESR (mm/h)		34 (15.00–65.75)^†^
RMD diagnoses	Arthritis (RA, JIA) (*n*, %)	130 (35.4)
	SLE (*n*, %)	55 (15.0)
	Vasculitides (IgA vasculitis, eosinophilic granulomatosis with polyangiitis, microscopic polyangiitis, granulomatosis with polyangiitis, Behçet, Buerger’s, Cogan, GCA, Takayasu) (*n*, %)	48 (13.1)
	PsA (*n*, %)	31 (8.5)
	Multiple RMD diagnoses (*n*, %)	27 (7.4)
	IIM (*n*, %)	25 (6.8)
	SSc (*n*, %)	27 (7.4)
	axSpA (*n*, %)	23 (6.3)
	Undiagnosed yet (*n*, %)	16 (4.4)
	pSS (*n*, %)	15 (4.1)
	Gout (*n*, %)	7 (1.9)
	APL (*n*, %)	5 (1.4)
	FMS (*n*, %)	4 (1.1)
	Autoinflammatory syndromes (TRAPS, FMF) (*n*, %)	3 (0.8)
	Sarcoidosis (*n*, %)	3 (0.8)
	REA (*n*, %)	2 (0.5)
	RPF (*n*, %)	2 (0.5)

*APL* antiphospholipid syndrome, *axSpA* axial spondyloarthritis, *BMI* body mass index, *CRP* C-reactive protein, *dL* deciliter, *ESR* erythrocyte sedimentation rate, *FMF* familial Mediterranean fever, *FMS* fibromyalgia syndrome, *g* grams, *GC* glucocorticoids, *GCA* giant cell arteritis, *hr* hour, *IgA* immunoglobulin A, *IIM* idiopathic inflammatory myopathies, *IV* intravenous, *JIA* juvenile idiopathic arthritis, *mg* milligrams, *mm* millimeters, *PsA* psoriatic arthritis, *pSS* primary Sjögren’s syndrome, *RA* rheumatoid arthritis, *REA* reactive arthritis, *RMDs* rheumatic musculoskeletal diseases, *RPF* retroperitoneal fibrosis, *SD* standard deviation, *SLE* systemic lupus erythematosus, *SSc* systemic sclerosis, *TRAPS* tumor necrosis factor receptor-associated periodic syndrome, *yrs* years. ^†^ Median (interquartile range, IQR)

To facilitate classification, related diagnoses were grouped into broader disease categories based on clinical features and pathophysiology. Rheumatoid arthritis (RA) and juvenile idiopathic arthritis (JIA) have been grouped together under the term “arthritis”. In a similar manner, vasculitis included IgA vasculitis, eosinophilic granulomatosis with polyangiitis (EGPA), microscopic polyangiitis (MPA), granulomatosis with polyangiitis (GPA), Buerger’s disease, Cogan syndrome, and giant cell arteritis (GCA). Idiopathic inflammatory myopathies (IIM) was used as an umbrella term for dermatomyositis, polymyositis and anti-synthetase syndrome. Tumor necrosis factor receptor-associated periodic syndrome (TRAPS) and familial Mediterranean fever (FMF) have been classified as autoinflammatory syndromes. Other diseases were maintained separately and include systemic sclerosis (SSc), systemic lupus erythematosus (SLE), psoriatic arthritis (PsA), axial spondyloarthritis (axSpA), Sjögren’s syndrome (pSS), gout, antiphospholipid syndrome (APL), and fibromyalgia (FMS). Blood parameter data for each patient were obtained from their respective medical records. The study was approved by the Larissa University Hospital Scientific Board (30/3rd/20-02-2025), and all participants provided informed consent.

### Data collection

Trained dietitians took anthropometric measurements following standardized procedures. Data collection included measurement of body weight using a digital floor scale (Kern MPE 200 K-1PEM, Kern, Germany) and height using a stadiometer (Seca 220, Hamburg, Germany). These measurements allowed calculation of body mass index (BMI) for all patients, which in turn categorized them into three groups: underweight (< 18.5 kg/m²), normoweight (≥ 18.5 kg/m² and < 25 kg/m²), and overweight/obesity (≥ 25 kg/m²).

### Orthorexia nervosa

The Düsseldorf Orthorexia Scale (DOS) is a validated questionnaire used to assess ON [33, 34]. The tool was translated into the Greek language using a standard forward–backward translation procedure. First, two bilingual translators (MGG and ECP) independently translated the original questionnaire into Greek. The two forward translations were compared, and a reconciled version was developed through discussion and resolution of discrepancies. This version was subsequently back-translated into English by an independent bilingual translator (AG), who was blinded to the original questionnaire. The back-translated version was then compared with the original instrument to ensure semantic and conceptual equivalence. Minor linguistic adjustments were made where necessary to enhance clarity and cultural appropriateness. The translation and use was permitted by the authors of the original DOS manuscript [33, 34]. The tool comprises of 10 items designed to assess orthorexic behaviors and attitudes, utilizing a four-point Likert scale, ranging from “this applies to me” (4 points) to “this does not apply to me” (1 point). The maximum possible score is 40, with greater scores indicating more pronounced orthorexic tendencies. A cutoff score of ≥ 30 suggests the presence of ON, while scores between 25 and 29 (corresponding to the 95th percentile) indicate an elevated risk for ON.

### Statistical analyses

Descriptive statistics summarized the characteristics of the study sample. Medians and interquartile ranges (IQR) were reported for continuous variables, while frequencies and percentages were calculated for categorical variables. The prevalence of the three DOS categories was calculated overall and stratified by each disease group to examine variations in disordered eating behavior across clinical populations. The internal consistency of the DOS was evaluated using Cronbach’s α, in accordance with established recommendations for the appropriate reporting and use of questionnaire-based research [35]. All analyses included only complete cases, excluding participants with missing data on variables relevant to each analysis.

To explore associations between independent variables and the DOS score, both simple and multiple linear regression analyses were conducted, treating the DOS score as a continuous outcome. Although ordinal regression models would typically be appropriate for ordered categorical outcomes like DOS categories, they were not employed due to a substantial imbalance in the distribution of responses. Most participants clustered within the first category, limiting the model’s ability to produce stable and interpretable estimates. Given this limitation, linear regression was selected as the most suitable analytical approach. For the multiple linear regression analyses, variables were selected using backward elimination based on the Akaike Information Criterion (AIC). This approach identifies the most parsimonious model, balancing fit and complexity, rather than relying solely on univariable significance. Statistical significance was defined as *p* < 0.05, and all analyses were conducted using R Studio (version 4.4.1) [36].

## Results

### Orthorexia nervosa and the DOS

**Fig. 1:**
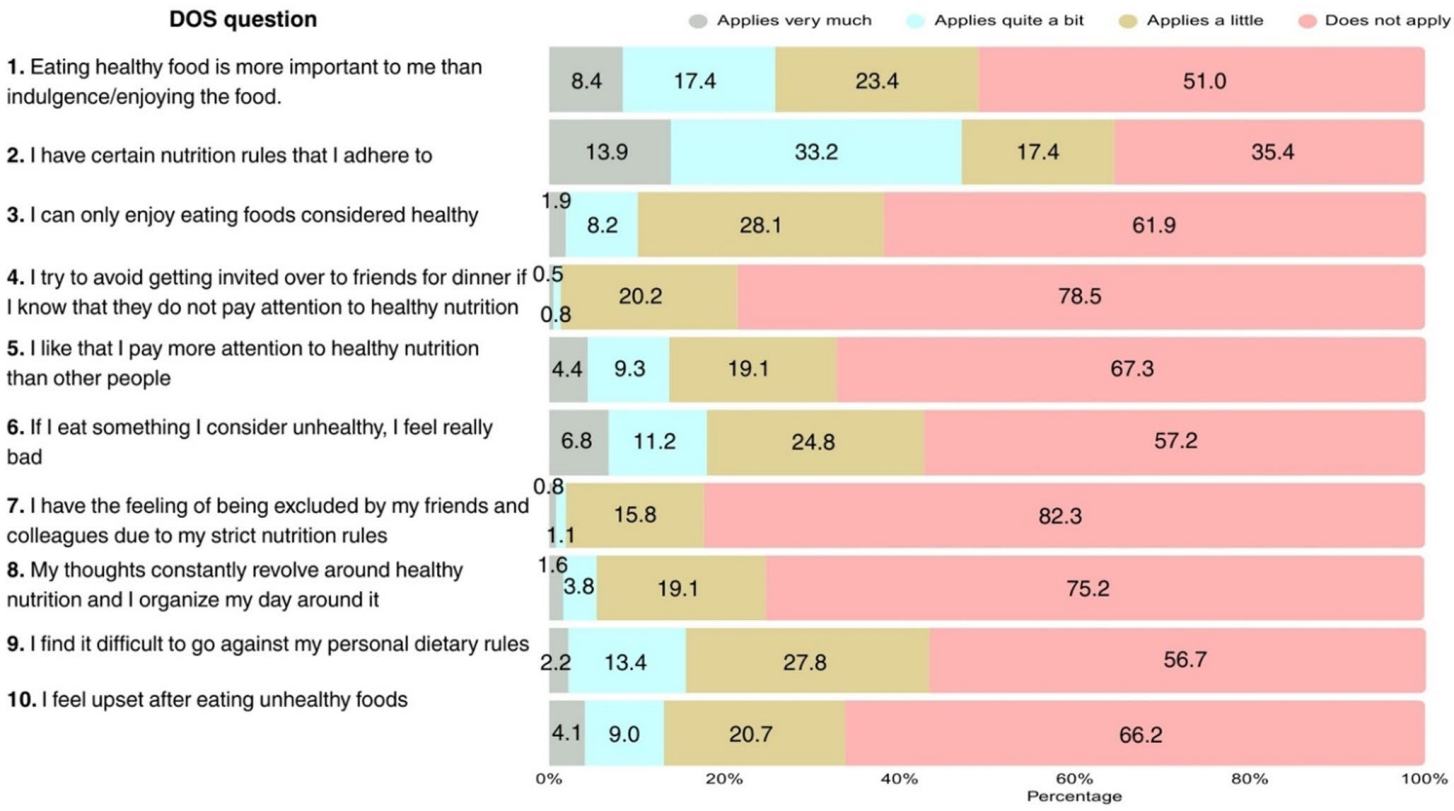
Response distribution for DOS items (*N* = 367). DOS: Düsseldorf Orthorexia Scale

 Figure 1 presents the distribution of participant answers in the DOS. The majority of patients, representing 93.2%, were identified as being free of ON-risk, 4.6% were classified as being at-risk for developing ON, and the remaining 2.2% screened positive for ON. The DOS demonstrated good internal consistency (Cronbach’s α = 0.89). Among the 10 questionnaire items, the statement most frequently endorsed was “I have certain nutrition rules that I adhere to,” with 13.9% of participants agreeing and 33.2% agreeing “quite a bit”. The second most endorsed statement involved “Eating healthy food is more important to me than indulgence/enjoying the food,” with 8.4% of the sample agreeing to the statement and 17.4% indicating it applied “quite a bit”.

A tendency to feel bad after consuming something perceived as unhealthy was reported by 18% of the sample, while 15.6% expressed difficulty in going against their personal dietary rules. A mere 13.7% of the sample reported paying more attention to healthy nutrition than other people.

The statements most strongly rejected by participants were “I have the feeling of being excluded by my friends and colleagues due to my strict nutrition rules” and “I try to avoid getting invited over to friends for dinner if I know that they do not pay attention to healthy nutrition,” with 98.1% of the sample expressing disagreement with both statements.

### ON in RMDs

**Fig. 2:**
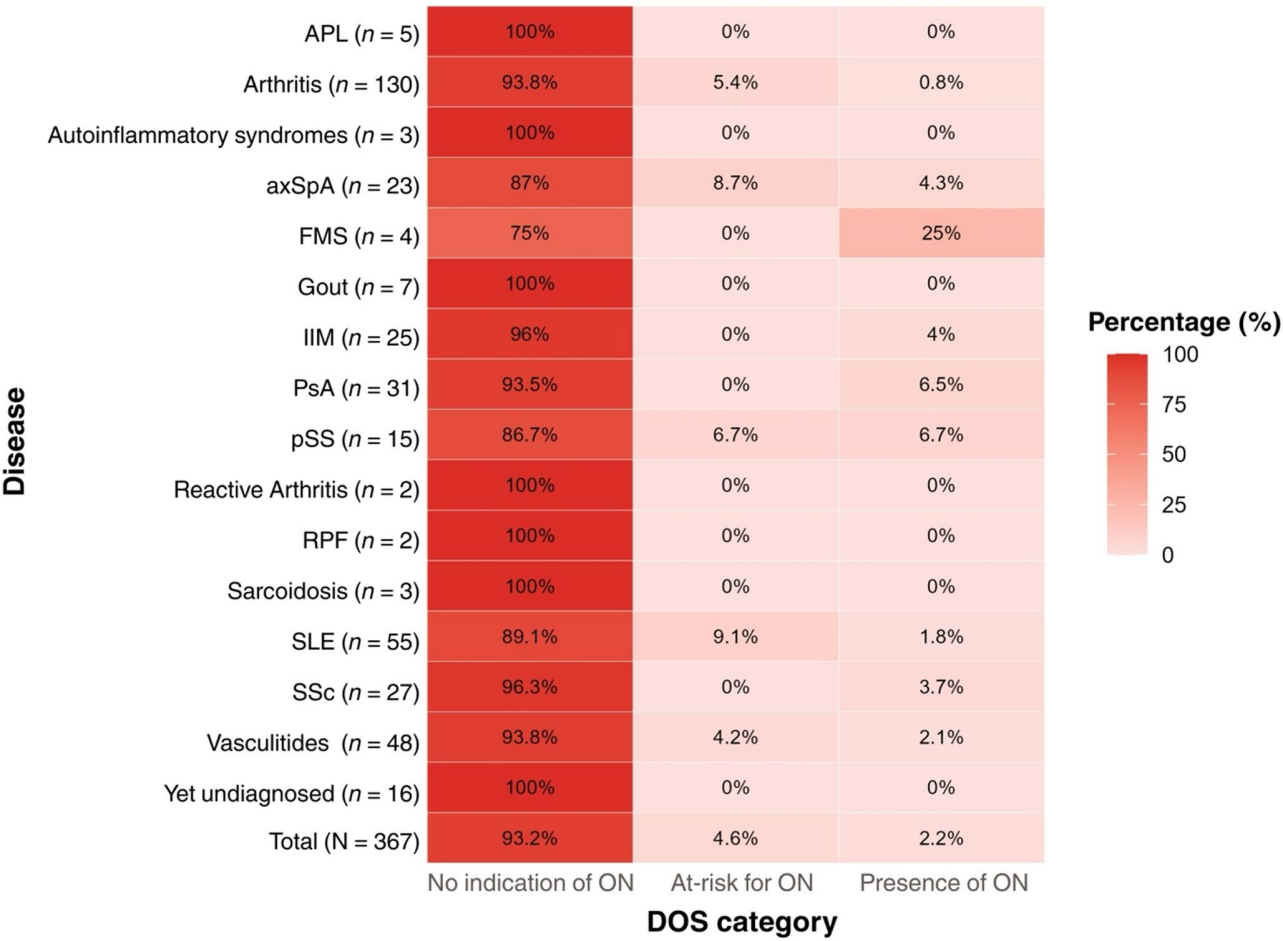
Prevalence of ON in patients with RMDs (*N* = 367). *APL* antiphospholipid syndrome; *axSpA* axial spondyloarthritis, *DOS* Düsseldorf Orthorexia Scale, *FMS* fibromyalgia syndrome, *ON* orthorexia nervosa, *PsA* psoriatic arthritis, *pSS* primary Sjögren’s syndrome, *RMDs* rheumatic and musculoskeletal diseases, *RPF* retroperitoneal fibrosis, *SLE* systemic lupus erythematosus, * SSc* systemic sclerosis

The prevalence of patients with RMDs screening positive for ON appears to vary across disease diagnoses. Among those presenting an orthorexic behavior (DOS ≥ 30), most had a diagnosis of fibromyalgia syndrome (FMS, 25%), 6.5% had PsA and primary Sjögren’s syndrome (pSS), 4.3% had an axSpA diagnosis, 4% had IIM, 3.7% suffered from SSc, and less than 4% had an SLE, vasculitis, or arthritis diagnosis. Of the patients in category 2, defined as being at-risk for ON based on the DOS, 9.1% had an SLE diagnosis, 8.7% were diagnosed with axSpA, 6.7% with pSS, 5.4% with arthritis, and 4.2% had some form of vasculitis. Details are presented in Fig. 2.

### Linear regression analyses

Simple linear regression analyses examined the association between individual demographic and clinical predictors and orthorexic tendencies, assessed using the DOS (Table 2). Among the demographic variables, female sex was associated with higher DOS scores (β = 2.41, 95% CI: 1.16, 3.65, *p* = 0.0002). Furthermore, a diagnosis of FMS was related to elevated orthorexic traits (β = 8.17, 95% CI: 2.61, 13.73, *p* = 0.004), while individuals with SLE also exhibited greater DOS scores (β = 2.01, 95% CI: 0.39, 3.64, *p* = 0.02). Conversely, IIM was associated with low DOS scores (β=-2.39, 95% CI: -4.70, -0.09, *p* = 0.04), indicating a potential negative association between the disease and orthorexic tendencies.

**Table 2:** Linear regression analyses of ON (Ν = 367)

Predictor	Beta-coefficient	95% (CI)	SE	*p* value	*R* ^2^	Adjusted *R*^2^
Age	-0.01	[-0.05, 0.02]	0.02	0.49	0.001	-0.001
BMI	-0.04	[-0,14, 0.06]	0.05	0.42	0.001	0.00
Women	2.41	[1.16, 3.65]	0.63	0.0002*	0.038	0.035
APL	-2.91	[-7.94, 2.12]	2.56	0.26	0.00	0.001
Autoinflammatory syndromes	0.332	[-6.15, 6.82]	3.30	0.92	0.00	-0.003
Current GC use	0.43	[-0.76, 1.62]	0.60	0.48	0.001	0.001
FMS	8.17	[2.61, 13.73]	2.82	0.004*	0.02	0.02
Gout	-3.74	[-7.99, 0.51]	2.16	0.08	0.00	0.005
IIM	-2.39	[-4.70, -0.09]	1.17	0.04*	0.01	0.009
PsA	0.47	[-1.08, 2.03]	0.79	0.65	0.00	0.001
pSS	2.57	[-0.37, 5.51]	1.5	0.09	0.00	-0.001
RA	0.15	[-1.07, 1.37]	0.62	0.81	0.00	-0.003
REA	-3.69	[-11.61, 4.23]	4.03	0.36	0.00	0.00
RPF	5.36	[-2.55, 13.27]	4.02	0.18	0.00	0.002
Sarcoidosis	1.341	[-5.14, 7.82]	3.30	0.68	0.00	-0.002
SLE	2.01	[0.39, 3.64]	0.83	0.02*	0.02	0.013
axSpA	0.68	[-1.73, 3.08]	1.23	0.58	0.00	-0.002
SSc	0.12	[-2.12, 2.35]	1.14	0.92	0.00	-0.003
Undiagnosed yet	-1.028	[-3.89, 1.83]	1.45	0.48	0.00	0.005
Vasculitides	-1.13	[-2.86, 0.60]	0.88	0.20	0.01	0.002

*APL* antiphospholipid syndrome, * axSpA* axial spondyloarthritis, *BMI* Body mass index, * CI* confidence intervals, * FMS* fibromyalgia syndrome, *GC* glucocorticoids, *IIM* idiopathic inflammatory myopathies, *ON* orthorexia nervosa, *PsA* psoriatic arthritis, *pSS* primary Sjögren’s syndrome, *RA* rheumatoid arthritis, *REA* reactive arthritis, *RPF* Retroperitoneal fibrosis, *SE* Standard error, *SLE* systemic lupus erythematosus, *SSc* systemic sclerosis; * *p* < 0.05

Other possible predictors, including participant age, BMI, glucocorticoid (GC) use, diagnosis of gout or other autoimmune RMDs (e.g., RA, PsA, SSc, vasculitides), presented lower levels of ON, with minimal explanatory power. A tendency towards significance was observed in gout and pSS diagnoses (*p* = 0.08 and *p* = 0.09, respectively).

### Multiple linear regression analyses

Two multiple linear regression models were performed to account for potential confounding variables. The first model (Table 3) included participant age, BMI, and sex, and revealed that women demonstrated higher orthorexic tendencies (β = 2.44, 95% CI: 1.19, 3.69, *p* = 0.0002) compared to the men.

**Table 3:** Multiple linear regression analyses of ON (Ν = 367)

Predictor	Beta-coefficient	95% (CI)	SE	*p* value	*R* ^2^	Adjusted *R*^2^
Age	-0.01	[-0.05, 0.03]	0.02	0.57	0.042	0.034
BMI	-0.04	[-0.13, 0.06]	0.05	0.49		
Women sex	2.44	[1.19, 3.69]	0.63	0.0002*		

*BMI* body mass index, *CI* confidence intervals, *ON* orthorexia nervosa, *SE* standard error; * *p* < 0.05

The second model (Table 4) included GC use, BMI, and disease duration. In this model, only disease duration was associated with ON (β = 0.06, 95% CI:0.01, 0.11, *p* = 0.028), suggesting that individuals with a more longstanding condition were more likely to develop ON.

**Table 4:** Multiple linear regression analyses of ΟΝ (Ν = 367)

Predictor	Beta-coefficient	95% (CI)	SE	*p* value	*R* ^2^	Adjusted *R*^2^
Current GC use	0.49	[-0.79, 1.78]	0.65	0.45	0.0175	0.086
BMI	-0.04	[-0.15, 0.06]	0.05	0.38		
Duration of disease diagnosis	0.06	[0.01, 0.11]	0.03	0.028*		

*BMI* body mass index, *CI* confidence interval, *GC* glucocorticoids, *ON* orthorexia nervosa, *SE* standard error, * *p* < 0.05

## Discussion

To our knowledge, this is the first study to screen for ON using the DOS in a large and diverse population of patients with RMDs. Among individuals living with RMDs, screening positive for ON was most commonly observed in those with FMS and SLE, particularly among women and those with longer disease duration. Conversely, individuals with IIM showed a reduced risk of ON, indicating a decreased prevalence of concerns pertaining to orthorexic behaviors.

There is a prevailing notion that autoimmunity is linked to eating disorders via shared biological pathways through the psychoneuroimmunological axis and underlying inflammatory processes [37, 38]. The presence of pain, physical dysfunction, and an impaired quality of life (QoL) has been identified as a contributing factor to the development of depression and social isolation [39]. Anxiety disorders have also been observed to be prevalent within this population [39], and consist of risk factors for an obsessive focus on healthy eating [40]. Moreover, the findings of this study indicated that while most patients were free of ON-risk, they adhered to stringent dietary regimens and prioritized health over the enjoyment of food, suggesting a tendency towards disordered thinking in relation to food.

FMS involves chronic musculoskeletal pain, joint and muscle stiffness, fatigue, and a range of psychological and cognitive symptoms, including mood disorders, cognitive dysfunction, anxiety, and depression [19, 41]. It affects women disproportionately [42], with growing evidence indicating that they are more likely to adhere to restrictive diets, avoid foods such as cereals, alcohol, and soft drinks, and prefer herbal products instead [43]. Concerns regarding body weight and physical appearance are also commonly reported, as are emotional eating and eating-related concerns [44, 45]. Furthermore, serum levels of brain-derived neurotrophic factor (BDNF) are negatively associated with hunger, thereby suggesting a potential correlation between eating patterns and dopaminergic activity in individuals with FMS [44]. In the present study, patients with FMS were found to be at higher risk for ON, which prompts further investigation into whether dietary rigidity in FMS represents a coping mechanism, a response to misinformation about “anti-inflammatory” diets, or an expression of underlying anxiety and perfectionism.

SLE primarily affects women and is associated with a broad range of clinical manifestations, such as renal, pulmonary and cardiac involvement [46]. Neuropsychiatric manifestations are observed in over half of patients with SLE [47], including symptoms such as psychosis, depression, anxiety disorders, and cognitive dysfunction [48, 49]. Although ON has not yet been systematically studied in this population, many individuals with SLE report favoring plant-based diets, reducing the consumption of animal products and processed foods, making dietary changes aimed at alleviating symptoms [50], and adhering to disordered eating [30]. Our findings indicate that patients with SLE exhibited greater DOS scores, suggesting an increased predisposition to orthorexic behaviors. These overall tendencies may reflect health-focused dietary control, but also raise important questions regarding the potential emergence of orthorexic behaviors in the context of chronic autoimmune disease.

Eating disorders appear relatively common among individuals with SLE, and recent genome-wide association studies (GWAS) have identified a shared genetic locus between autoimmune disorders and AN, underscoring the role of genetic predisposition in this comorbidity [30, 51]. Several case reports and case series have documented the occurrence of AN in patients with SLE [51] and juvenile-onset SLE (jSLE) [30], with the majority of reported cases involving girls and young women. Beyond genetics, chronic inflammation emerges as another shared pathway, as both conditions are characterized by elevated levels of pro-inflammatory cytokines [37, 38]. Furthermore, brain-reactive autoantibodies identified in SLE have been shown to disrupt neurotransmitter systems involved in appetite regulation [51]. Within this framework, AN in the setting of SLE could plausibly be regarded as a neuropsychiatric manifestation of SLE, driven primarily by immune dysregulation and inflammatory mechanisms rather than being solely a side effect of treatment [30, 51]. In the context of a post-SLE diagnosis, the clinical manifestations of autoimmune disease, in conjunction with treatment-related changes such as weight gain induced by corticosteroids and altered appearance, may have the potential to act as triggers for psychosocial stressors [31, 51].

Inflammatory myopathies consist of heterogeneous diseases that affect primarily the skeletal muscle, along with other organs, and lead to myalgia and weakness [52]. These manifestations often contribute to a reduced QoL in patients with IIM, who frequently report low energy levels and increased social isolation [53]. In addition to these challenges, physical impairments and elevated rates of depression appear to play a significant role in further lowering QoL scores [53]. A particularly impactful factor influencing QoL is dysphagia, which is highly prevalent among patients with IIM [52, 54]. This swallowing disorder can cause various difficulties, potentially leading to serious complications such as aspiration pneumonia and unintended weight loss [52]. Consequently, individuals encountering such challenges may struggle to maintain a balanced diet and may not prioritize strict ‘clean’ eating, instead focusing on foods that are less likely to worsen swallowing issues. This may be particularly relevant when interpreting the absence of increased orthorexic risk observed among patients with IIM.

Altered body perception has been reported more frequently among women [31], and ON appears to follow the same trend [55]. In the context of RMDs, women were found to be at-risk for ON [32], a finding that was also supported by the present study. This pattern may be partly explained by changes in body image [31], which can stem from RMD-related alterations in physical appearance, disease flares, and greater functional limitations [56]. These factors may contribute to heightened body awareness and more rigid dietary attitudes. Moreover, the fear of deterioration and relapse is a characteristic feature of chronic diseases [15]. In this context, many patients develop a strong desire to prevent or manage their condition by adopting a healthy, whole-food diet [57, 58], which may be relevant to the orthorexic patterns exhibited by individuals with a prolonged disease course.

Nevertheless, while studies in the general population report a high prevalence of orthorexic behaviors —sometimes affecting more than half of individuals [59]– such tendencies appear to be far less common among patients with RMDs. This contrasts sharply with other chronic conditions, such as diabetes mellitus [11], inflammatory bowel disease [14], and celiac disease [12], as well as with lifestyle groups that follow highly restrictive eating patterns, such as vegetarianism [60]. In these conditions, strict dietary modifications are often perceived as central to symptom control and disease management, resulting in a strong emphasis on nutrition and a willingness to adopt highly restrictive diets. In RMDs, however, disease management primarily relies on pharmacological interventions, with dietary modification being considered as complementary, rather than essential. Nutritional advice in these disorders is typically supportive and advisory, rather than prescriptive or mandatory [61], leaving less room for the development of rigid and perfectionistic attitudes toward food. Furthermore, the burden of chronic pain, fatigue, physical disability and reduced QoL that frequently accompanies RMDs [39] may limit both the motivation and the practical ability to adhere to highly structured and restrictive dietary routines.

However, orthorexic tendencies warrant careful consideration, specifically within the setting of RMDs. Research has demonstrated that chronic illness can precipitate cognitive and psychological vulnerability [62]. While patients with RMDs didn’t screen positive for ON at elevated rates, certain diagnoses showed a higher frequency of orthorexic behaviors. This highlights the need for a cautious and balanced approach to food and dietary patterns, especially when these are promoted as complementary strategies in disease management. Several dietary interventions—such as the Autoimmune Protocol (AIP) diet—have been suggested to help alleviate symptoms of autoimmune diseases [58]. However, these approaches, which run in parallel to ON, often involve the elimination of multiple food groups and, if undertaken without the guidance of a qualified nutrition professional might lead to nutrient deficiencies [58]. As a consequence, they may contribute to the development of disordered eating behaviors, an unhealthy relationship with food [58], and nutritional deficits, or over-restriction that may worsen disease outcomes [27, 28]. Nevertheless, the repercussions of ON impact extend beyond the physical domain, encompassing the social and psychological domains; they reverberate beyond physical health, eroding psychological well-being, straining social relationships, and ultimately diminishing overall QoL [63]. Furthermore, such tendencies have the potential to diminish adherence to treatment regimens, favoring ‘natural’ dietary methods over pharmacotherapy and consequently resulting in an unfavorable disease progression. Given the significance of various metabolic conditions such as malnutrition [64], sarcopenia [65], and rheumatoid cachexia [66] in the context of RMDs, the emergence of ON may further exacerbate these implications. Thus, dietary recommendations in patients with RMDs should be personalized, evidence-based, and accompanied by appropriate clinical and psychological support.

### Limitations of the study

This study is not without limitations. This is a cross-sectional analysis, in essence; therefore, it is not possible to draw causal relationships. Moreover, the incorporation of a control group would have been advantageous in enhancing the quality of the study and reinforcing the findings. ON screening was conducted using the DOS [33, 34], as no diagnostic tools have been validated and universally accepted for ON assessment. Moreover, the grouping of certain diseases into more general categories is conducive to the prevention of loss of statistical power; however, it is important to note that this may result in a limitation of valuable insights. Also, actual dietary practices were not assessed, which could have provided a more comprehensive background of the recorded orthorexic tendencies. Similarly, variables such as depression, anxiety, and body image were beyond the scope of the present study and thus, not recorded, although they might have offered additional context. Linear regression analyses were performed instead of ordinal regression models, due to the disproportionate distribution of participants in the first DOS category. This may have affected the ability to capture the full ordinal nature of the outcome variable.

## Conclusion

Orthorexic tendencies have been observed within patients with RMDs, particularly among women, those with longer-standing disease, and those diagnosed with FMS and SLE. Although the risk for ON in this population appears relatively low, its potential impact on disease outcome and severity is significant. Despite the limited research in this area, emerging evidence suggests that genetic and inflammatory factors could be associated with an increased risk of eating disorders in related diseases, such as SLE. Disordered eating behaviors driven by an excessive preoccupation with “healthy” eating can contribute to nutritional deficiencies, increased disease activity, and poorer QoL. Given these risks, there is an urgent need for more rigorous studies focusing on orthorexia within specific subgroups of patients with RMDs. Such research should aim to identify individuals at risk, elucidate the underlying psychological and social factors contributing to these tendencies, and develop targeted interventions to prevent extreme behaviors that may jeopardize patients’ health and well-being.

## Data Availability

The datasets collected for this manuscript are accessible from the corresponding author upon reasonable request.
